# Intracolonic Administration of the TRPA1 Agonist Allyl Isothiocyanate Stimulates Colonic Motility and Defecation in Conscious Dogs

**DOI:** 10.1007/s11605-015-2813-4

**Published:** 2015-04-09

**Authors:** Soutoku Someya, Munenori Nagao, Chikashi Shibata, Naoki Tanaka, Hiroyuki Sasaki, Daisuke Kikuchi, Tomohiro Miyachi, Takeshi Naitoh, Michiaki Unno

**Affiliations:** Department of Surgery, Tohoku University Graduate School of Medicine, 1-1 Seiryo-machi, Aoba-ku Sendai, 980-8574 Japan

**Keywords:** Allyl isothiocyanate, Giant migrating contractions, TRPA1

## Abstract

**Background:**

The aim of the present study was to investigate the effects of the intracolonic transient receptor potential (TRP) A1 agonist allyl isothiocyanate (AITC) on colonic motility and defecation.

**Methods:**

The effects of AITC administered into the proximal colonic lumen on colonic motility and defecation were studied in neurally intact dogs equipped with strain-gauge force transducers on the colon, with or without various antagonists. Effects of intracolonic AITC were also studied in dogs with either transection/re-anastomosis (T/R) between the proximal and middle colon and complete extrinsic denervation of an ileocolonic segment.

**Results:**

AITC increased colonic motility and induced giant migrating contractions (GMCs) with defecations in 75 % of experiments in neurally intact dogs. These effects were inhibited by atropine, hexamethonium, ondansetron, and HC-030031 but unaltered by capsazepine. In dogs with T/R, the increase in colonic motility was inhibited in the middle-distal colon. In dogs with extrinsic denervation, the increase in colonic motility in the distal colon was decreased.

**Conclusions:**

Intracolonic AITC stimulates colonic motility and defecation via cholinergic, serotonergic, and TRPA1 pathways. Continuity of colonic enteric neurons plays an essential role in the intracolonic AITC-induced colonic motor response, while extrinsic nerves are important in occurrence and propagation of GMCs.

## Introduction

Transient receptor potential (TRP) channels are a series of cation channels classified into seven subfamilies: TRPA, TRPC, TRPL, TRPM, TRPN, TRPP, and TRPV.^1^^,^^2^ TRP channels are expressed in many tissues and have various physiologic functions, including thermo-sensation. TRPA1 is activated by cold temperatures less than 17 °C and by the major pungent ingredient of wasabi allyl isothiocyanate (AITC).^3^ Recently, many studies on TRPA1 have been performed, but most of them are related to the central nervous system and respiratory system.^4^^,^^5^ Very few reports have been published on the effect of intraluminal administration of AITC on gastrointestinal motility, despite the fact that AITC is an ingredient of a spice.

Two types of colonic contractions occur in conscious dogs; one is colonic motor complexes (CMCs), which consist of a tonic increase of the baseline pressure with superimposing phasic contractions.^6^^–^8 Most CMCs occur in the proximal colon and propagate distally with a slow velocity (4 cm/min). CMCs are thought to be associated with slow “to and fro” movement of the intracolonic contents. Another type of colonic contractile activity is giant migrating contractions (GMCs) with high amplitude and a much faster propagation velocity (1 cm/s).^6^^,^^9^ Because defecation is observed when GMCs reach the distal colon, GMCs are thought to be related to the rapid transit of feces and defecation.^6^^,^^9^^,^^10^ We reported previously that intracolonic capsaicin induced GMCs and defecation immediately after its administration, and continuity of colonic enteric neurons and extrinsic nerves play an important role in the intracolonic capsaicin-induced increase in colonic motility.^11^^,^^12^ Capsaicin is a pungent ingredient of chili and is an agonist for TRPV1.^11^ Other than capsaicin, hot temperatures greater than 43 °C or acid stimulate TRPV1.^3^ Because both TRPV1 and TRPA1 are present in the muscle, nerves, and along the entire gastrointestinal tract,^13^^,^^14^ it is likely that intracolonic administration of the TRPA1 agonist AITC would increase colonic motility, like capsaicin. AITC at dose of 1 mg/kg administered orally in dogs induced GMCs at the distal colon.^13^

Based on these findings, we had two hypotheses: (1) intracolonic AITC would stimulate colonic motility and induce GMCs and defecation, and (2) extrinsic innervation to the colon and continuity of intrinsic neurons within the colon wall play a role in the intracolonic AITC-induced motor response. The aim of the current study was to investigate the effect of intracolonic AITC on colonic motility and defection, and to investigate its mechanism of action in conscious dogs. The effects of AITC to stimulate colonic motility and defecation have possibility that this agent might be of use clinically as a prokinetic drug for the colon.

## Material and Methods

### Preparation of Animals

Procedures and animal care were performed according to the guidelines of the Animal Care and Use Committee of the Tohoku University. All surgical preparations were performed using sterile techniques. In all, 15 beagle dogs (body weight 11–13 kg) were divided into the following three groups: neurally intact dogs (*n* = 5), dogs with transection/re-anastomosis (T/R) between the proximal and middle colon (*n* = 5), and dogs with complete extrinsic denervation of an ileocolonic segment (*n* = 5).

Anesthesia was induced by intravenous sodium thiopental (20 mg/kg; Ravonal; Mitsubishi Tanabe Pharm Co., Osaka, Japan) and maintained by inhaled sevoflurane (Sevofrane; Abbott Japan Co., Osaka, Japan) with oxygen. Before laparotomy, we placed a silicone catheter (SH No. 2; Create Medic Co., Yokohama, Japan) into the superior vena cava via the right jugular vein in all three groups to administer drugs.

Via a middle celiotomy, three strain-gauge force transducers (F-12IS; Star Medical Inc., Tokyo, Japan) were sewn onto the seromuscular surface of the colon to measure contractile activity of the circular muscle. One transducer (C1) was placed on the proximal colon 5 cm distal to the ileocolonic junction, another (C3) was implanted at the distal colon 10 cm proximal to the peritoneal reflection, and third (C2) was implanted on the middle colon so that the distance between each colonic transducer would be equal. A silicone catheter to administer test materials into the proximal colonic lumen was positioned via the cecum with its tip at C1. The lead wires from the transducers and silicone catheters were tunneled subcutaneously and exteriorized via a stab wound between the scapulae. The lead wires and the catheters were covered with a canvas jacket for protection from self-inflicted trauma.

**Fig. 1:**
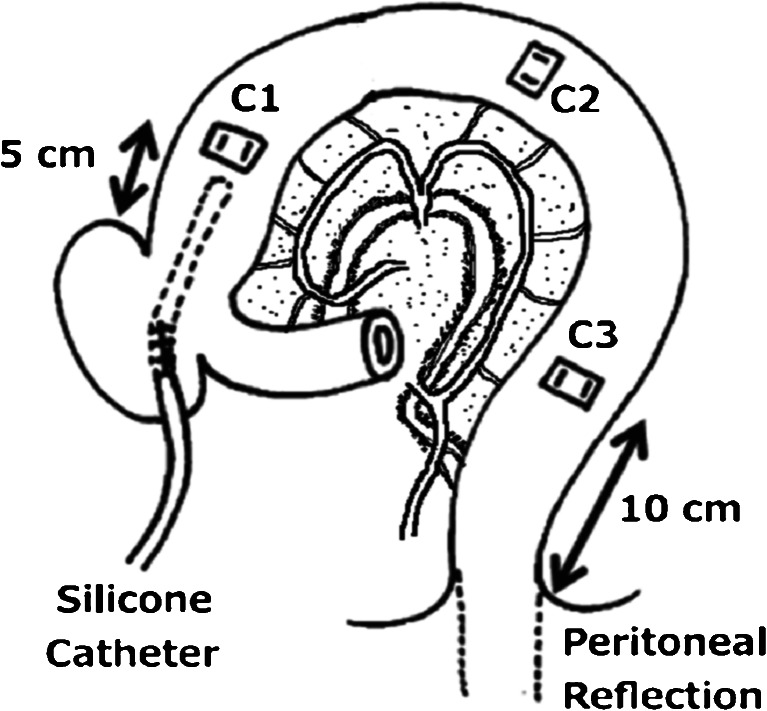
Canine preparation. The first (*C1*) and third (*C3*) transducers were placed on the proximal colon 5 cm distal to the ileocolic junction and 10 cm proximal to the peritoneal reflection, respectively. The second transducer (*C2*) was implanted midway between *C1* and *C3*. A silicone catheter was positioned in the proximal colonic lumen via the cecum. Transection and re-anastomosis (T/R) was performed midway between *C1* and *C2*. In dogs with extrinsic denervation, all extrinsic nerves were transected by removing all tissues maintaining continuity between the segment and the dog except for arteries and veins feeding the segment from the ileum to the distal colon

These procedures were performed in all three groups. No other surgical procedures were performed in neurally intact dogs. In dogs with T/R, the colon was transected at the midpoint between C1 and C2, and colonic continuity was restored by an immediate end-to-end sutured anastomosis. In dogs with extrinsic denervation, all extrinsic nerves were transected by removing all tissues maintaining continuity between the segment and the dog except for the arteries and veins feeding the segment from the ileum 7 cm proximal to the ileocolonic junction to the distal colon 10 cm proximal to the peritoneal reflection (Fig. 1). All dogs were allowed to recover for 14 days after the operation. Colonic motility was then monitored in the conscious state by connecting the lead wire from the transducers to an amplifier (MS-08; Star Medical Inc.), and then to a computer with a software program (Chart; AD Instruments, Victoria, Australia) through Mac Lab (AD Instruments). The dogs were fed a solid meal (CD-55a; Clea Japan Inc., Tokyo) once a day in the evening. Water was given ad libitum except for the duration of the experiments.

### Experimental Protocol

The dogs were fasted for 16 h before each experiment. In neurally intact dogs, 10 mL of 154 mmol/L NaCl solution (Otsuka Pharmaceutical Factory, Inc., Tokushima, Japan) containing AITC (5 or 10 mg) was administered as a bolus into the colonic lumen through the silicone catheter when all colonic transducers were in the quiescent period. The intestinal motor response induced by 10 mL of 154 mmol/L NaCl solution, which contained 2.0 mL of dimethyl sulfoxide as the vehicle for AITC, was used as a placebo control. To study the mechanism of the AITC-induced colonic motor response, the following antagonists or placebo control were given intravenously 5 min before the intracolonic AITC (10 mg): muscarinic antagonist atropine (0.1 mg/kg), nicotinic antagonist hexamethonium (5 mg/kg), 5-hydroxytryptamine-3 (5-HT_3_) receptor antagonist ondansetron (0.5 mg/kg). Intracolonic administration of the TRPA1 antagonist HC-030031 (4 mg), or the TRPV1 antagonist capsazepine (5 mg) was performed 5 min before intracolonic administration of AITC (10 mg) from the silicone catheter. Doses of these antagonists were determined according to previous reports.^11^^,^^12^^,^^15^

In dogs with T/R or with extrinsic denervation, the effect of intracolonic AITC at the doses of 10 mg on colonic motility was compared to that in neurally intact dogs. All experiments started 14 days after T/R or extrinsic denervation and were finished by 28 days after T/R or extrinsic denervation.

All experiments were carried out in a random order and repeated twice except for studies using the antagonist in which experiments were performed once. The mean of the two studies was regarded as a representative value for that dog. AITC was administrated only once per day.

### Data Analysis

The occurrence of GMCs was analyzed by a visual inspection. GMCs were defined as single-event contractions with a mean amplitude greater than 2.8 times greater than that of CMCs and propagating with a mean velocity of about 1.0 cm/s.^7^ If defecation or GMCs were observed within 30 min after administration of agents, then these were regarded to be agent induced. The area under the contractile curve measured for 30 min after administration of each solution was expressed as a motility index (MI). A commercial computer program (Chart; AD Instruments) was used to calculate the MI. The Mann–Whitney *U* test and the chi-square test were used to compare the MI and the occurrence of defecation or GMCs, respectively. All data were expressed as the mean ± standard error of the mean (SEM), and values of *p* < 0.05 were regarded to be significant.

### Drugs

AITC, atropine, hexamethonium, ondansetron, and capsazepine were purchased from Wako Pure Chemical Industries, Ltd., Osaka. HC-030031 was purchased from Funakoshi Co, Ltd., Tokyo.

## Results

### Effect of Intracolonic Administration of AITC in Neurally Intact Dogs

**Fig. 2:**
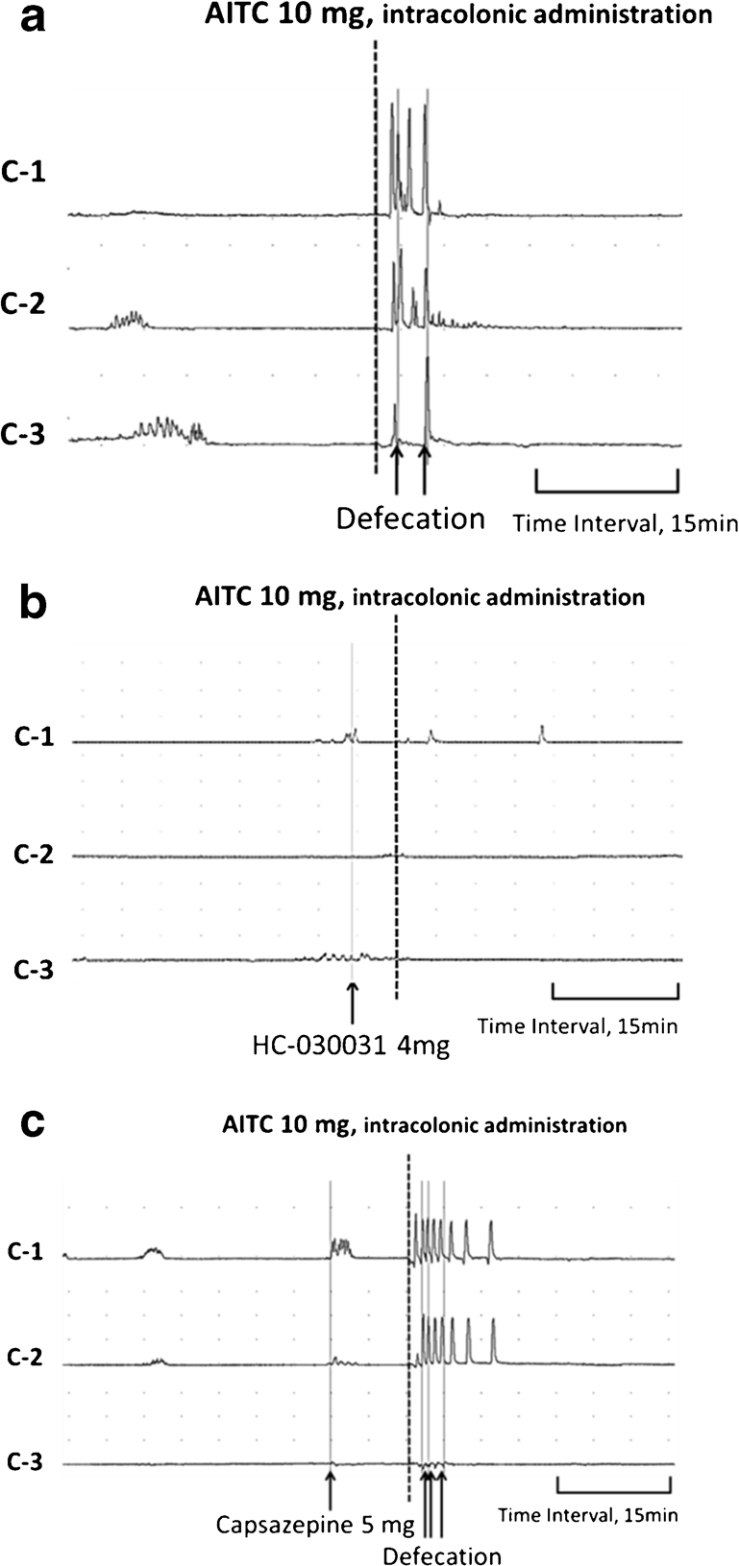
Representative tracings showing the effects of intracolonic AITC on colonic motor activity in neurally intact dogs. **a** Intracolonic AITC at the dose of 10 mg evoked GMCs in the colon and induced defecation. In this tracing, four GMCs were observed in the proximal colon, two of which reached the distal colon and induced defecations. **b** Pretreatment with intracolonic HC-030031 5 min before intracolonic AITC abolished the intracolonic AITC-induced motor response. **c** The effects of intracolonic AITC was not altered by pretreatment with intracolonic capsazepine

**Fig. 3:**
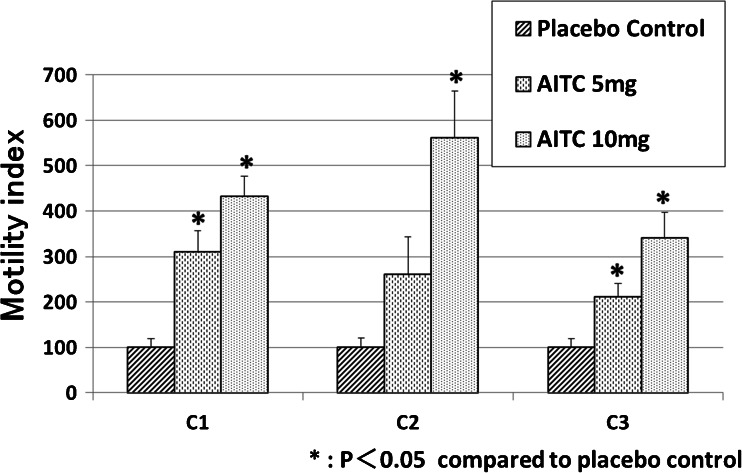
Response of the motility index (MI) in response to two doses of intracolonic AITC in neurally intact dogs. AITC at the doses of 5 mg increased the MI compared with placebo control at *C1* and *C3*. AITC at doses of 10 mg increased the MI compared with placebo control at *C1*, *C2*, and *C3*. *p < 0.05 compared with placebo control

The intracolonic administration of the placebo control had no effect on colonic motility. Intracolonic AITC at doses of 5 and 10 mg caused GMCs in 7 and 10 of 10 experiments, and induced defecations in 6 and 9 of 10 experiments, respectively (Table 1, Fig. 2a). Although AITC did not induce GMCs in 3 of 10 experiments with the 5-mg dose, CMCs were observed in those 3 experiments. AITC increased the MI compared with placebo control at the C1 and C3 at the 5-mg dose and at C1–C3 at the 10-mg compared to placebo control (*p* < 0.05, Fig. 3).

**Table 1:** Rate of GMCs and defecation in response to various doses of intracolonic AITC in neurally intact dogs

	GMCs	Defecation
Placebo control	0/10	0/10
AITC 5 mg	7/10*	6/10*
AITC 10 mg	10/10*	9/10*

*p < 0.05 compared to placebo control

### Effect of Various Antagonists on the AITC-Induced Motor Response in Neurally Intact Dogs

**Fig. 4:**
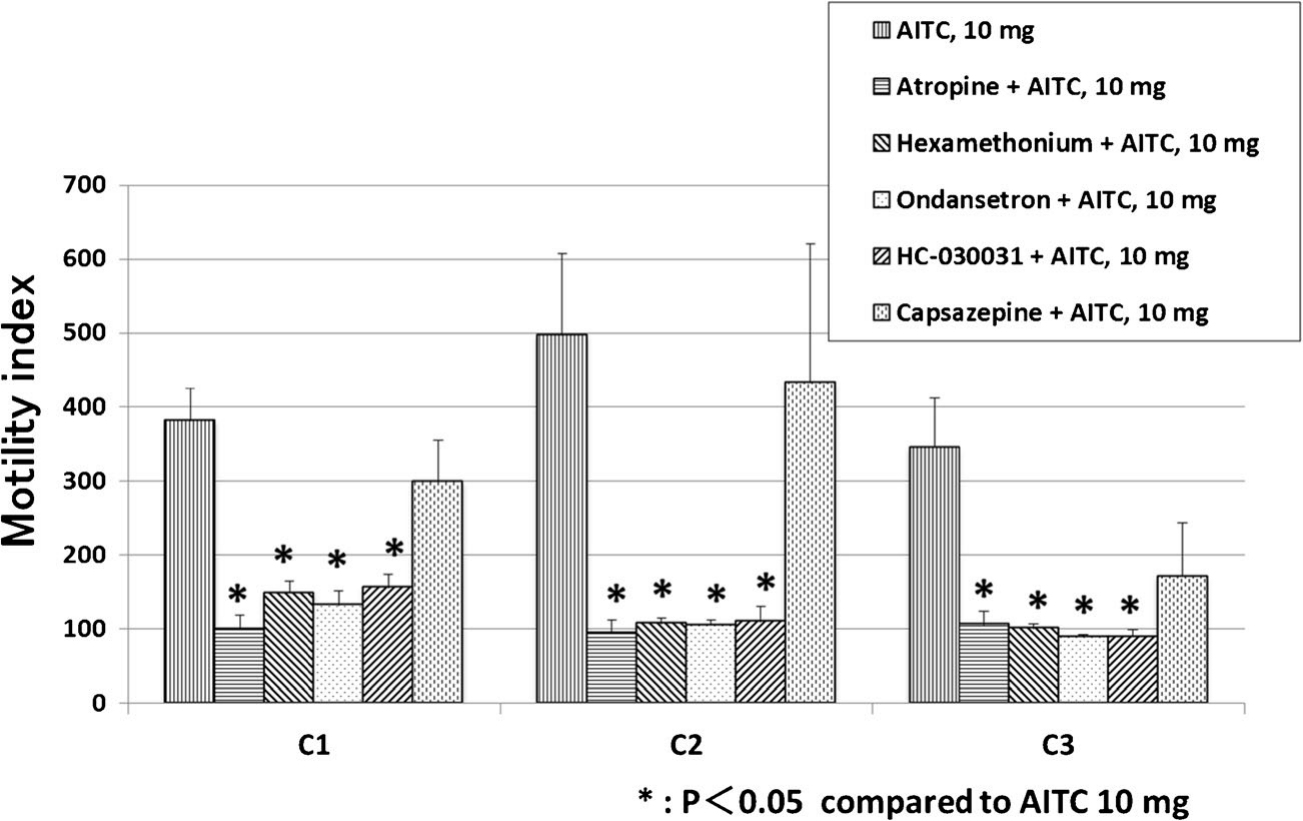
Effect of various antagonists on the intracolonic AITC-induced increase in MI. Atropine, hexamethonium, ondansetron, and HC-030031 inhibited the intracolonic AITC-induced increase in MI at all sites. Capsazepine did not affect the intracolonic AITC-induced increase in MI. *p < 0.05 compared with placebo control

Figure 4 shows the effect of various antagonists on the AITC-induced increase in the MI. Atropine, hexamethonium, ondansetron, and HC-030031 abolished the intracolonic AITC-induced motor response at all sites (Figs. 2b and 4) and inhibited the occurrence of GMCs and defecations (Table 2). Pretreatment with capsazepine did not obviously affect the colonic motor response nor the GMCs induced by AITC (Fig. 2c, Table 2), but the number of defecations was decreased in dogs pretreated with capsazepine compared to AITC 10 mg (Table 2).

**Table 2:** Rate of intracolonic AITC-induced GMCs and defecations in the presence of various antagonists in neurally intact dogs

	GMCs	Defecation
AITC 10 mg	5/5	5/5
Atropine + AITC 10 mg	0/5*	1/5*^a^
Hexamethonium + AITC 10 mg	0/5*	1/5*^a^
Ondansetron + AITC 10 mg	0/5*	0/5*
HC-030031 + AITC 10 mg	1/5*	0/5*
Capsazepine + AITC 10 mg	3/5	2/5*

*p < 0.05 compared to AITC 10 mg

^a^Defecation not associated with GMCs

### Effect of AITC in Dogs with T/R and Extrinsic Denervation

**Fig. 5:**
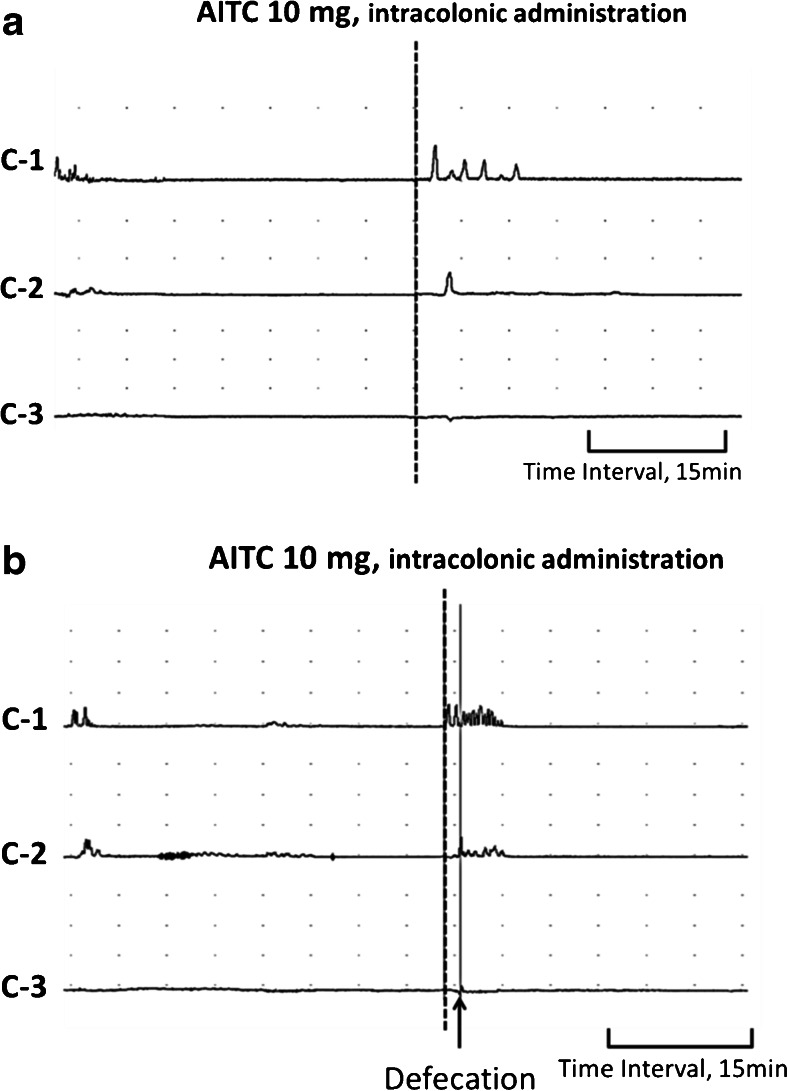
A representative tracing showing the effects of intracolonic AITC on colonic motility in dogs with T/R (**a**) and in dogs with extrinsic denervation (**b**). **a** In dogs with T/R, contractions which did not meet criteria of GMCs or CMCs occurred at *C1*, but they rarely propagated to the middle colon across the anastomosis. **b** In dogs with extrinsic denervation, AITC induced CMCs in the proximal-middle colon

**Fig. 6:**
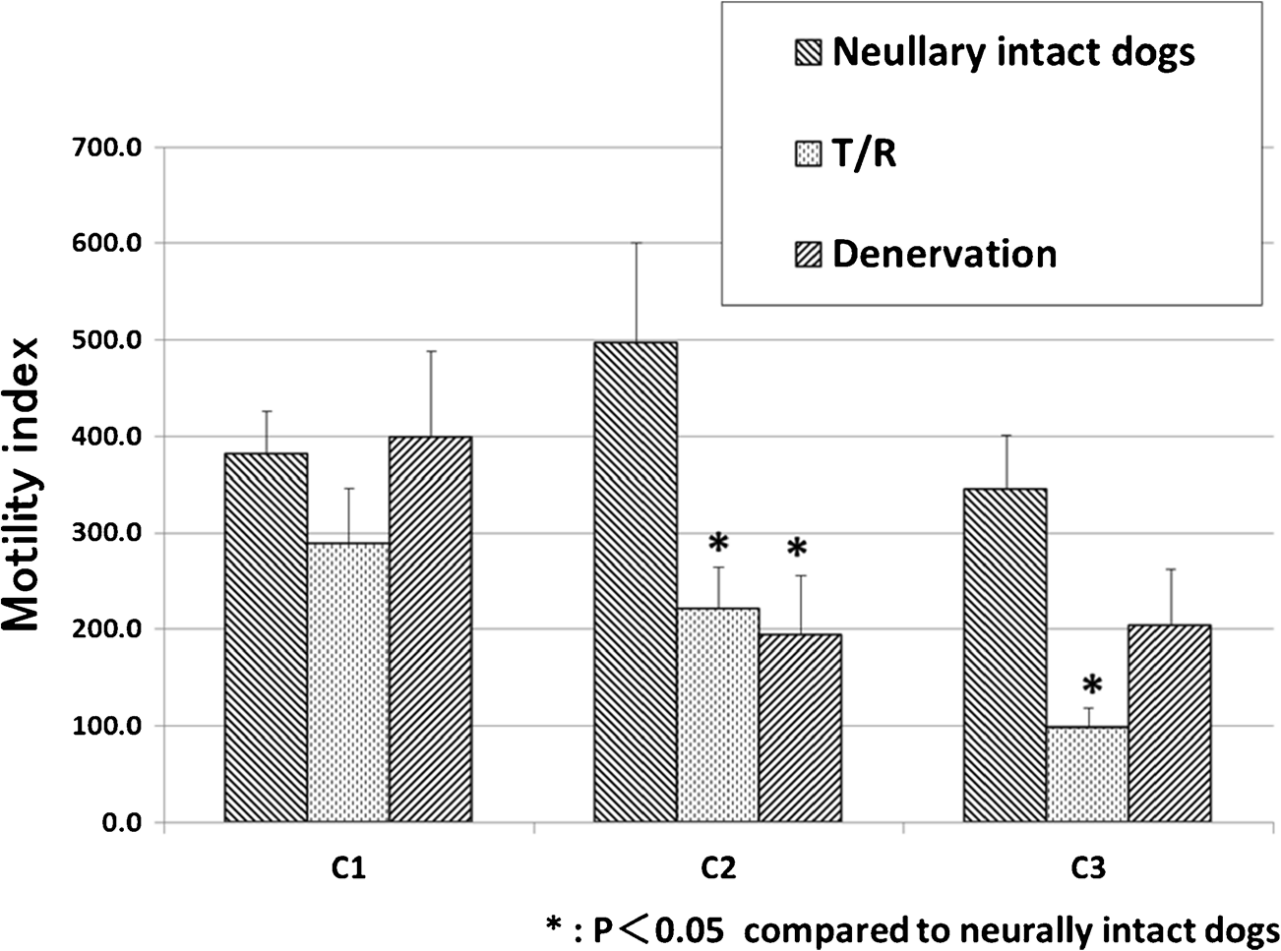
MI response to intracolonic AITC at doses of 10 mg in dogs with T/R and extrinsic denervation. The MI at *C2*–*C3* but not at *C1* in dogs with T/R was decreased compared to neurally intact dogs. In dogs with extrinsic denervation, the MI at *C1* and *C3* did not differ from neurally intact dogs, but the MI at *C2* was decreased. *p < 0.05 compared with neurally intact dogs

In dogs with T/R, intracolonic AITC at a dose of 10 mg caused GMCs in 2 of 10 experiments, CMCs in 4 experiments, and contractions which did not meet the criteria of GMCs or CMCs in 4 experiments (Fig. 5a). The number of GMCs and defecations in dogs with T/R was decreased compared to neurally intact dogs (*p* < 0.05, Table 3). One of 3 defecations in dogs with T/R had no accompanying GMCs. Intracolonic AITC-induced contractions were mainly observed at C1 in dogs with T/R (Fig. 5a). As a result, the MI at C1 in dogs with T/R did not differ from neurally intact dogs, but the MI at C2 and C3 was decreased (*p* < 0.05, Fig. 6).

**Table 3:** Rate of intracolonic AITC-induced GMCs and defecations in dogs with T/R and extrinsic denervation

	GMCs	Defecation
Neurally intact dogs	10/10	9/10
Dogs with T/R	2/10*	3/10*
Dogs with denervation	4/10*	9/10

*p < 0.05 compared to neurally intact dogs

In dogs with extrinsic denervation, intracolonic AITC at a dose of 10 mg caused GMCs in 4 of 10 experiments, CMCs in 4 experiments (Fig. 5b), and contractions which did not meet definition of GMCs or CMCs in 2 experiments. The number of AITC-induced GMCs in dogs with extrinsic denervation was decreased compared to neurally intact dogs (*p* < 0.05, Table 3). Because defecations not associated with GMCs were identified in 5 experiments (Fig. 5b), 9 defecations were observed in 10 experiments. Thus, frequency of defecation in dogs with extrinsic denervation did not differ from neurally intact dogs (Table 3). AITC mainly induced colonic contractions at C1 in dogs with denervation (Fig. 5b). Compared to neurally intact dogs, the MI at C1 and C3 in dogs with extrinsic denervation did not differ significantly, but the MI at C2 was decreased (Fig. 6).

## Discussion

This study demonstrated that intracolonic administration of the TRPA1 agonist AITC increased colonic motility and induced GMCs and defecation. This intracolonic AITC-induced colonic motor response and defecation appear to be mediated via muscarinic, nicotinic, and 5-HT_3_ receptors, because the responses were inhibited by atropine, hexamethonium, and ondansetron. These observations for AITC were similar to the intracolonic capsaicin-induced colonic motor response and defecation.^11^ We used a specific TRPA1 antagonist HC-030031^3^ and showed that the inhibitory effects of HC-030031 were similar to atropine, hexamethonium, and ondansetron. We performed experiments using capsazepine, a specific antagonist for TRPV1, because of the possibility that AITC might evoke its effects not only via TRPA1 but also via TRPV1. In our study, pretreatment with capsazepine did not inhibit the intracolonic AITC-induced colonic motor response but decreased the number of defecations. These results with HC-030031 and capsazepine suggest that intracolonic AITC increases colonic motility and induces defecations mainly via TRPA1. The possibility remains, however, that the doses of AITC used in the present study stimulated TRPV1 as well.

The effect of intracolonic AITC on colonic motility was also studied in dogs with several forms of localized denervation T/R and extrinsic denervation. Dogs with T/R are model for disruption of continuity of the colonic enteric neurons. We studied the effect of intracolonic AITC on colonic motility beginning 14 days after T/R, and we assume that continuity of the intramural nerves was not re-established by then. It took 4–8 weeks for reconnection of intrinsic neurons after T/R in guinea pigs.^16^

In dogs with T/R, the colonic motility index was decreased in the middle and distal colon but not in the proximal colon, and the frequency of GMCs and defecations was decreased compared to neurally intact dogs. In contrast, in dogs with extrinsic denervation, the colonic motility index was decreased only in the middle colon, and the frequency of GMCs was decreased compared to neurally intact dogs. In dogs with extrinsic denervation, the frequency of defecations did not differ from neurally intact dogs, and defecations not accompanied by GMCs were observed frequently; this finding was an obvious difference between dogs with T/R and denervation. These results suggest that continuity of intrinsic enteric neurons play an essential role in the intracolonic AITC-induced increase in colonic motility, GMCs, and defecations, whereas extrinsic innervation of the colon plays a role mainly in the occurrence and propagation of GMCs.

TRPA1 is expressed at the sensory nerve terminals and enterochromaffin cells of the gastrointestinal tract.^13^ TRPA1 expression in the colon has been shown in several species including rats, mice, and dogs.^13^^,^^17^^,^^18^ In dogs, oral administration of AITC evoked contractions in the gastric antrum, jejunum, and colon and induced defecations.^13^ Therefore, AITC induced contractions both locally and distantly in the gastrointestinal tract. Considering our present and previous results together, stimulation of the TRPA1 with intracolonic AITC appears likely to induce proximal colonic contractions, either directly or indirectly. The increase in proximal colonic contractile patterns that propagate caudally along the intrinsic nerves is associated with rapid transit of intraluminal contents distally in the colon. Rapid transit of feces is related to GMCs, and when GMCs reach the distal colon, defecations are observed. Extrinsic nerves are necessary for the occurrence and the organized migration of GMCs. We reported similar results previously when studying the effect of the intracolonic TRPV1 agonist capsaicin on colonic motility and defecation in dogs.^11^^,^^12^ The effects of intracolonic administration of AITC on colonic motility has not been studied so far. Our findings in the present study contribute to the better understanding of regulation of colonic motility via afferent fibers in the colon, especially role of extrinsic nerves and continuity of intrinsic nerves in the enhancement of colonic motility induced by stimulation to afferent nerves in the proximal colon.

Defecations not accompanied by GMCs were frequently observed in dogs with extrinsic denervation in the present study. In neurally intact dogs, GMCs occurred in the proximal-middle colon and reached the distal colon whenever defecations were observed. Thus, GMCs are considered to be closely associated with the mass movement and evacuation of feces. Defecations were always accompanied by GMCs in dogs with extrinsic denervation of the rectum, while in dogs with rectal T/R or extrinsic denervation of the rectum plus rectal T/R, defecations occurred without GMCs.^19^ These previous results seem to be in conflict with our current study which showed that defecations without GMCs were frequently observed in dogs with extrinsic denervation of the colon but were observed rarely in dogs with T/R. This difference might be due to the different regions of T/R and extrinsic denervation between their study and ours. The effect on colonic motility in these two studies was greater for T/R rather than extrinsic denervation. We previously reported that intravenous injection of the α-2 adrenoceptor antagonist yohimbine induced defecations accompanied by GMCs in conscious dogs.^20^ In dogs with extrinsic denervation of the ileocolon, yohimbine often induced GMCs without defecations, and defecations were observed very infrequently. These results of our previous study differ from our present study in which defecations without GMCs often occurred in dogs with extrinsic denervation. We believe that this difference was related to the different routes of stimuli and involved receptors; the effects in the present study were mediated via TRPA1 at the sensory nerve endings which were stimulated by intracolonic AITC, while yohimbine given intravenously stimulated colonic motility by antagonizing α-2 adrenoceptors via hematogenous delivery.

One of limitations in this study is that we did not confirm the completeness of surgical extrinsic denervation. We previously used this method of denervation,^11^ and the same method was also performed in the small intestine by other investigators.^21^ In that previous report, they found the catecholamine levels in the denervated segment to be unmeasurable; this result indicates the validity of surgical extrinsic denervation. We believe that obviously different motor response to intracolonic AITC between neurally intact and extrinsically denervated dogs is caused by the extrinsic denervation.

The effects of AITC in the present study might be of use clinically as a prokinetic drug for the colon. Doihara et al. reported that intragastric administration of AITC increased colonic motility and stimulated defecations in dogs, but vomiting was evoked concurrently by intragastric AITC.^13^ This effect of intragastric AITC to induce vomiting is a major disadvantage in terms of clinical application. The same phenomena were observed for intragastric and intracolonic administration of capsaicin.^9^^,^^22^ Therefore, development of an oral drug which would dissolve in the proximal colonic lumen might lead to clinical application of AITC as a prokinetic agent.
